# Which Risk Factors and Colposcopic Patterns Are Predictive for High-Grade VAIN? A Retrospective Analysis

**DOI:** 10.3390/diagnostics13020176

**Published:** 2023-01-04

**Authors:** Anna Daniela Iacobone, Davide Radice, Maria Elena Guerrieri, Noemi Spolti, Barbara Grossi, Fabio Bottari, Sara Boveri, Silvia Martella, Ailyn Mariela Vidal Urbinati, Ida Pino, Dorella Franchi, Eleonora Petra Preti

**Affiliations:** 1 Preventive Gynecology Unit, European Institute of Oncology IRCCS, Via Ripamonti 435, 20141 Milan, Italy; 2 Department of Biomedical Sciences, University of Sassari, 07100 Sassari, Italy; 3 Division of Epidemiology and Biostatistics, European Institute of Oncology IRCCS, 20141 Milan, Italy; 4 Department of Obstetrics and Gynecology, Luigi Sacco Hospital, ASST-Fatebenefratelli-Sacco, University of Milan, 20157 Milan, Italy; 5 Division of Laboratory Medicine, European Institute of Oncology IRCCS, 20141 Milan, Italy; 6 Laboratory of Biostatistics and Data Management, Scientific Directorate, IRCCS Policlinico San Donato, San Donato Milanese, 20097 Milan, Italy

**Keywords:** high-grade vaginal intraepithelial neoplasia (VAIN), smoking, previous hysterectomy for CIN2+, colposcopic grade, papillary lesion, vascular pattern

## Abstract

Colposcopic patterns of Vaginal Intraepithelial Neoplasia (VAIN) are not definitively related to histological grade. The aim of the present study was to investigate any correlation between clinical and colposcopic features and the development of high-grade VAIN. Two hundred and fifty-five women diagnosed with VAIN (52 VAIN1, 55 VAIN2 and 148 VAIN3) at the European Institute of Oncology, Milan, Italy, from January 2000 to June 2022, were selected for a retrospective analysis. Multivariate logistic regression was performed to estimate the association of risk factors and colposcopic patterns with VAIN grade. Smoking was associated with the development of VAIN (34.1%, *p* = 0.01). Most women diagnosed with VAIN3 (45.3%, *p* = 0.02) had a previous history of hysterectomy for CIN2+. At multivariate analysis, colposcopic grade G2 (OR = 20.4, 95%CI: 6.67–61.4, *p* < 0.001), papillary lesion (OR = 4.33, 95%CI: 1.79–10.5, *p* = 0.001) and vascularity (OR = 14.4, 95%CI: 1.86–112, *p* = 0.01) were significantly associated with a greater risk of VAIN3. The risk of high-grade VAIN should not be underestimated in women with a history of smoking and previous hysterectomy for CIN2+, especially when colposcopic findings reveal vaginal lesions characterized by grade 2, papillary and vascular patterns. Accurate diagnosis is crucial for an optimal personalized management, based on risk factors, colposcopic patterns and histologic grade of VAIN.

## 1. Introduction

Vaginal Intraepithelial Neoplasia (VAIN) is a rare premalignant lesion of the female lower genital tract, approximately 100-fold less common than cervical squamous intraepithelial lesions [1,2,3], with an estimated incidence of 0.2–2 per 100,000 women/year [4,5]. The prevalence of VAIN has recently increased due to improvements in screening methods, such as cytology and Human Papillomavirus (HPV) testing [5].

HPV infection is the fundamental etiological factor for the development of VAIN. However, other risk factors have been identified and investigated over time, including young age at first intercourse, a large number of sexual partners, cigarette smoking, immunosuppression, past or concurrent diagnosis of cervical or vulvar preinvasive or invasive lesions, previous hysterectomy for cervical intraepithelial neoplasia (CIN) or cervical cancer, prior radiotherapy, and a history of in utero exposure to diethylstilbestrol [6,7,8]. Recently, more attention has also been paid to the potential role of the vaginal microbiota, whose composition is influenced by hormonal status and changes during the development and progression of VAIN [9]

According to the depth of vaginal epithelium involved by dysplasia, VAIN is usually classified into grades 1, 2 or 3. The 2014 WHO classification of VAIN replaced the previous three-tiered system and recognizes only two categories: low-grade VAIN (VAIN1 or vaginal low-grade squamous intraepithelial lesion, LSIL) and high-grade VAIN (VAIN2-3 or vaginal high-grade squamous intraepithelial lesion, HSIL) [10,11]. VAIN1 is the result of a transient low-risk (LR) or high-risk (HR) HPV infection, with a high rate of spontaneous regression within 2 years. High-grade VAIN is due to a persistent and transforming HR-HPV infection and has a higher potential for recurrence and progression towards invasive vaginal carcinoma [12]. Since the risk of progression of VAIN2 to invasive cancer is still under discussion and should be intermediate between VAIN1 and VAIN3, some authors still consider VAIN2 as a separate category [13,14].

VAIN mostly occurs in women over 60 years of age, who are commonly asymptomatic but sometimes report vaginal discharge or bleeding [15]. Furthermore, post-menopausal women may be at increased risk of VAIN due to Lactobacillus depletion, overgrowth of anaerobic species and increased frequency of bacterial vaginosis, which have been indicated as agents responsible for delayed HPV clearance and subsequent carcinogenic progression [9,16].

The diagnosis is usually made by colposcopic-guided biopsy of suspicious vaginal lesions. After an abnormal cervical screening test with no lesion identified on the cervix, great attention should be paid to the complete evaluation of the vagina. Vaginal colposcopy is quite challenging, often due to vaginal dystrophy in post-menopausal women. In addition, colposcopic patterns of VAIN are highly heterogeneous and not very specific, thus resulting in a lack of correlation between colposcopy and histology, unlike CIN [17,18]. Nevertheless, few previous studies have investigated the potential link between colposcopic findings and the histopathologic grade of VAIN, in order to improve the predictive role of the colposcopic examination for treatment management [19,20].

The aim of the present study was to identify the potential risk factors for the development of VAIN to evaluate the diagnostic accuracy of colposcopy in relation to the histological grade of VAIN and to investigate any correlation between clinical and colposcopic features and high-grade VAIN.

## 2. Materials and Methods

All women affected by VAIN and who were attending the Preventive Gynecologic Unit of the European Institute of Oncology, Milan, Italy, from January 2000 to June 2022, were retrieved from hospital file archives and selected for a retrospective analysis.

The local Institutional Review Board approved the study protocol (IEO protocol UID 3821, date of approval: 27 October 2022) and written formal consent for the use of data for scientific purposes was signed by each subject.

Patients were included if the following criteria were met: (a) age at diagnosis of 25 years or older; (b) colposcopic-guided vaginal biopsies because of an abnormal pap smear or a previous history of any HPV-related lower genital tract diseases; (c) histologic confirmation of any grade of VAIN, including VAIN3 with stromal microinvasion; (c) available data about colposcopic findings. Patients were excluded in the case of (a) denied informed consent; (b) negative histology; or (c) diagnosis of invasive vaginal carcinoma.

Data regarding sociodemographic, clinical, laboratory and pathological characteristics of patients were recorded in a dedicated database.

Colposcopies were performed by staining with a 5% acetic solution and a 3% Lugol’s solution (Schiller test), by expert colposcopists working at the Preventive Gynecologic Unit of the European Institute of Oncology. Abnormal colposcopic findings were described as grade 1 if minor (thin acetowhite epithelium, fine punctuation, fine mosaic) or grade 2 if major (dense acetowhite epithelium, coarse punctuation, coarse mosaic), according to the 2011 Colposcopic Terminology of the International Federation for Cervical Pathology and Colposcopy (IFCPC) [21,22]. All records of colposcopies performed before the introduction of the 2011 IFCPC Colposcopic Terminology were revised accordingly. Location of the lesion (vaginal vault, upper, middle and/or lower thirds), and uni/multifocality, vascular and papillary (defined as an acetowhite exophytic lesion not to be misdiagnosed as condyloma) patterns were reported separately.

Single or multiple colposcopic-guided biopsies were taken from suspicious vaginal lesions with the worst colposcopic characteristics. Dedicated gynecological pathologists working at the Pathology Division of our Institute performed all pathologic diagnoses. In the case of multifocal lesions and different grades of VAIN, the worst pathologic diagnosis and the related colposcopic pattern was considered for our analysis.

When possible, the Cobas 4800 HPV test (Cobas; Roche Diagnostics), an HR-HPV DNA assay with concurrent partial genotyping, was performed on liquid-based cervical (LBC) specimens at the time of colposcopy. The Cobas test is a Real-Time PCR-based assay able to detect HR-HPV genotypes 16 and 18 in separate channels, as well as a group of 12 other HR-HPV types (31, 33, 35, 39, 45, 51, 52, 56, 58, 59, 66 and 68) in another channel. It is a fully automated test and includes an internal control (B-globin) as a marker of sample adequacy.

### Statistical Analysis

Categorical patients’ characteristics at diagnosis were summarized by counts and percent, age by mean and standard deviation and cross-tabulated by VAIN grade. Between VAIN grade groups, comparisons were done by using Fisher’s exact test for categorical variables and the F-test for age (one-way analysis of variance). Lesion type and vascularity were significantly associated with colposcopic grade and then entered two separated multivariate logistic regression analyses in order to estimate their association with VAIN grade as risk factors. Results are presented as Odds Ratios (OR) with 95% Confidence Intervals (CI). All tests were two-tailed and considered significant at the 5% level. All analyses were done using SAS 9.4 (Cary, NC, USA).

## 3. Results

After applying inclusion and exclusion criteria, 255 women affected by VAIN and attending the Preventive Gynecologic Unit of the European Institute of Oncology, Milan, Italy, from January 2000 to June 2022, were selected for our retrospective analysis.

VAIN 1, 2 and 3 were diagnosed in 52, 55 and 148 women, respectively.

The main clinical characteristics of patients are summarized by VAIN grade at diagnosis in Table 1.

The mean age of women at first diagnosis was 52.4 ± 12.8 years, with no significant difference among patients diagnosed with different histological grade of VAIN. About a third of cases was a current or former smoker (34.1%, *p* = 0.01) and more than half of patients reported previous pregnancies (55.4%, *p* = 0.02). Both variables were significantly associated with the diagnosis of VAIN, even according to histological grade.

Previous hysterectomy for CIN2+ was reported by 38.0% of women affected by VAIN, especially VAIN3 (45.3%, *p* = 0.02). Prior cervical cancer occurred in 49 patients undergoing hysterectomy (3 squamocellular carcinoma and 46 adenocarcinoma, *p* = 0.16), diagnosed as FIGO stage IA, IB and IIA-B in 29.4, 52.9 and 17.7% of cases (*p* = 0.17), respectively.

There was a significant correlation (*p* < 0.001) between cytology and histological grade of VAIN: 59.1% of VAIN1 were preceded by ASCUS-LSIL, whereas 73.1% of VAIN3 by ASCH-HSIL.

No other clinical variables, including immunosuppression, hormonal therapy, prior diagnosis of cervical or other cancers, previous or concomitant CIN, HPV-related VIN (vulvar intraepithelial neoplasia) or AIN (anal intraepithelial neoplasia), were significantly associated with VAIN and histological grade.

Interestingly, the HPV test was positive for HR-HPV with 16 and/or 18 and other HR-HPV not 16–18 in 44.4% and 39.4% of patients, respectively. Most women affected by VAIN3 were positive for HR-HPV with 16 and/or 18 (53.5%), however borderline significant (*p* = 0.05), probably due to the large number of missing data regarding the Cobas result in our population (*n* = 95).

Colposcopic findings in relation to the histological grade of VAIN are detailed in Table 2, including 10 VAIN3 with stromal microinvasion in the VAIN3 category.

**Figure 1:**
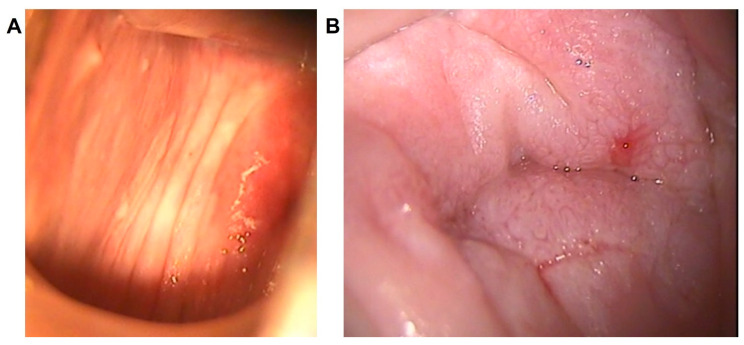
Colposcopic grade according to the 2011 IFCPC Colposcopic Terminology. (**A**): Grade 1 or minor, as shown by the colposcopic pattern (thin acetowhite epithelium) of a patient diagnosed with VAIN1, located at the upper third of the right vaginal wall. (**B**): Grade 2 or major, as shown by the colposcopic pattern (coarse mosaic) of a patient diagnosed with VAIN3, located at the vaginal vault, after previous hysterectomy for CIN3.

**Figure 2:**
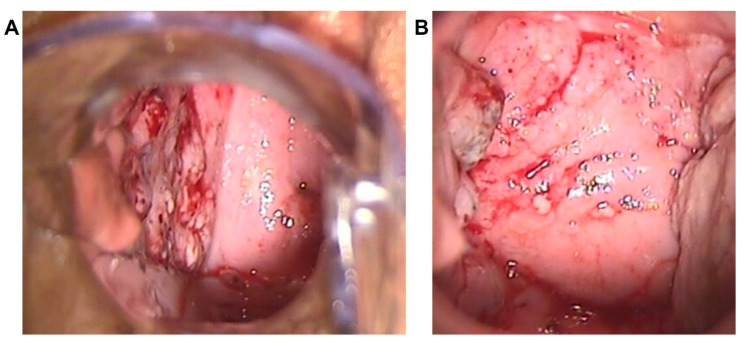
Papillary pattern. Multiple papillary lesions with both regular and irregular surface, located at the whole right vaginal wall in a patient diagnosed with VAIN3 at a colposcopic overview (**A**) and at a magnified colposcopic vision (**B**).

**Figure 3:**
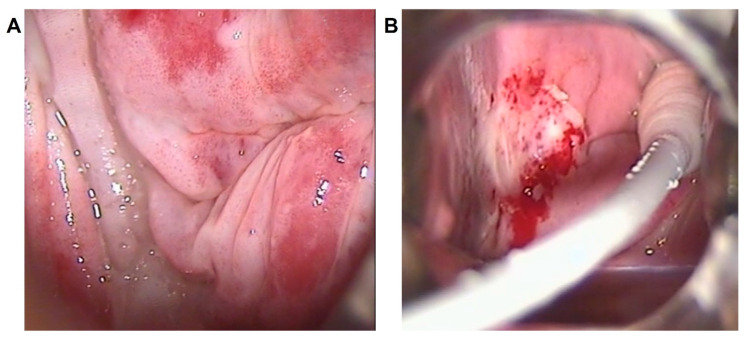
Vascular pattern. Colposcopic findings showing: dense acetowhite epithelium with coarse punctuation at the posterolateral right vaginal fornices and upper-third walls in a young woman (35 years) affected by VAIN3 (**A**); and dense acetowhite epithelium with fragile vessels at the upper-middle third of the right vaginal wall in a post-menopausal woman (62 years) affected by VAIN2 (**B**).

Most VAIN1 (88.2%) were characterized by colposcopic grade G1, and most VAIN3 (66.9%) were characterized by colposcopic grade G2 (*p* < 0.001) (Figure 1). A flat lesion was detected in 80.4% of VAIN1, whereas a papillary lesion was in 52.9% of VAIN3 (*p* < 0.001) (Figure 2). A vascular pattern was present in only 19.7% of VAIN, but there was a significant linear correlation according to histological grade (*p* < 0.001). Indeed, about one-third of VAIN3 (31.4%) showed a vascular pattern (Figure 3).

No significant association was found for multifocal lesions (*p* = 0.36) and vaginal localization (*p* = 0.09) by VAIN grade at diagnosis.

**Figure 4:**
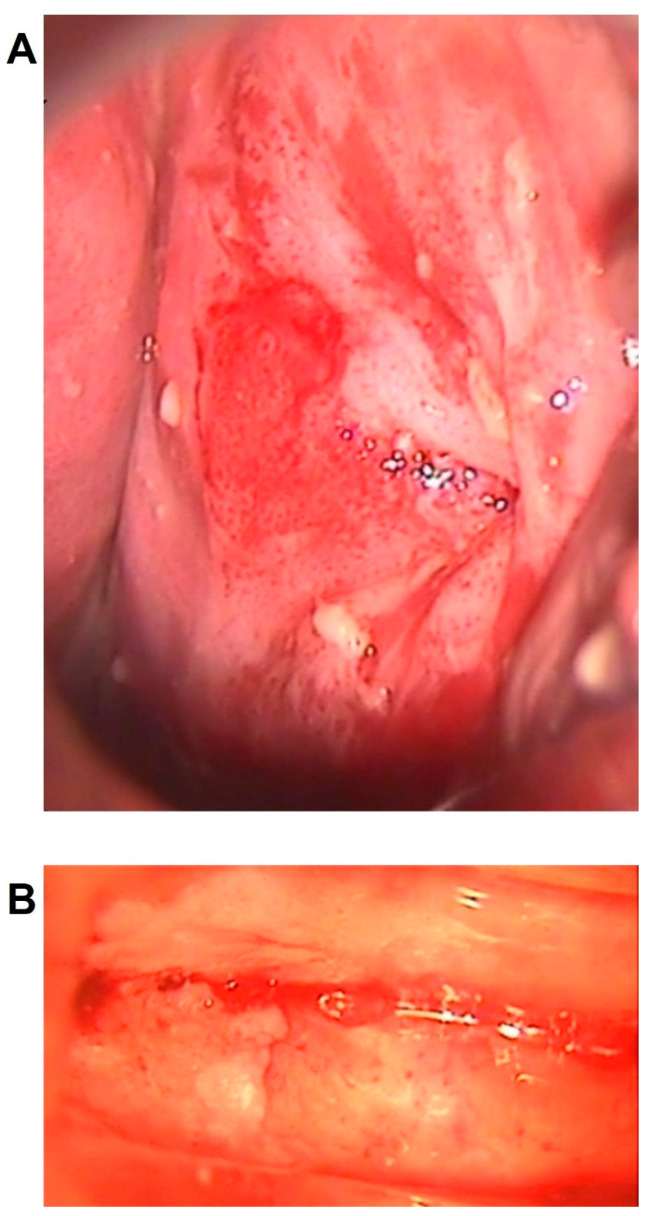
Colposcopic patterns associated with microinvasive VAIN3. Abnormal colposcopic findings of grade 2, vascular patterns and papillary lesions with regular (**A**) and/or irregular (**B**) surface in two women affected by VAIN3 with stromal microinvasion, both located at the vaginal vault, after previous hysterectomy for cervical cancer.

When considered as a separate category, VAIN3 with stromal microinvasion was significantly associated with colposcopic grade G2 (100%), papillary lesions (90.0%) and vascular pattern (44.4%) with a *p*-value <0.001 for all variables, as shown in Table 3 (Figure 4).

However, a sensitivity analysis showed that these colposcopic variables were still statistically significantly correlated with VAIN grade even after excluding all cases with microinvasive VAIN3 (Table S1).

Old medical reports of women firstly diagnosed with VAIN farther away from current times had some missing clinical and colposcopic data. The distribution of missing data by VAIN grade at diagnosis was evaluated as a possible selection bias for different variables (Table S2). Only the distributions of missing data for parity (*n* = 22, *p* = 0.03) and vascular pattern (*n* = 11, *p* = 0.02) were significant. In particular, all missing data regarding vascular pattern were in VAIN3 category. However, the arbitrary imputation of the missing data to the presence of the vascular pattern did not change the significance of the association with the histological grade of VAIN (data not shown).

Since lesion type and vascularity were significantly associated with colposcopic grade (Table S3), two separated multivariate logistic regression analyses were performed in order to estimate their association with high-grade VAIN as risk factors.

As shown in Table 4, at multivariate analysis, colposcopic grade G2 was significantly associated with a greater risk of developing both VAIN 2 (OR = 4.77, 95%CI: 1.40–16.2, *p* = 0.01) and VAIN3 (OR = 20.4, 95%CI: 6.67–61.4, *p* < 0.001).

When excluding the colposcopic grade from the multivariate logistic regression (Table 5), only papillary lesion represented a predictive factor for VAIN2 (OR = 2.90, 95%CI: 1.07–7.89, *p* = 0.03), whereas a previous hysterectomy for CIN2+ (OR = 2.37, 95%CI: 1.02–5.36, *p* = 0.04), papillary lesion (OR = 4.33, 95%CI: 1.79–10.5, *p* = 0.001) and vascular pattern (OR = 14.4, 95%CI: 1.86–112, *p* = 0.01) significantly led to a higher risk of VAIN3.

## 4. Discussion

Our findings confirmed that smoking, parity, previous hysterectomy for CIN2+ and abnormal cytology should be considered as potential risk factors for VAIN, and a significant association is maintained by histologic grade. In addition, abnormal colposcopic findings, including grade G2, papillary and vascular patterns, are predictive of the development of high-grade VAIN, even at multivariate analysis.

According to our results, current or former smoking was significantly associated with the risk of VAIN, as already well-known in previous literature [23,24,25]. Sherman et al. also showed that smoking is significantly associated with the occurrence of high-grade VAIN in women infected by HR-HPV [6], as a possible consequence of a biological interaction between smoke and the viral protein of HR-HPV genotypes. Due to the large number of missing data regarding HR-HPV status, it was not possible to investigate the same correlation in our study population.

In our analysis, parity was related with a significantly increased risk of developing VAIN and, in particular, high-grade VAIN, as opposed to previous findings [26]. However, it was not possible to exclude a selection bias due to the significant distribution of missing data for parity by VAIN grade at diagnosis.

It is well-established that women with a previous history of CIN or cervical cancer, who underwent hysterectomy, remain at a higher life-time risk of VAIN and should be carefully screened for HPV-related vaginal and vulvar disease throughout their lives [26,27]. Our study confirmed that prior hysterectomy for CIN2+ should be considered as a risk factor for high-grade VAIN. Indeed, VAIN after hysterectomy usually arises near the vaginal cuff [7], since HPV infection is often multifocal and may affect other sites of the female lower genital tract. Moreover, the grade of VAIN may be affected by the severity of previous cervical disease [26] and women with a history of CIN2+ should be extensively counselled regarding the future risk of VAIN before hysterectomy. Previous hysterectomy for HPV-related cervical lesions has also been recognized as a risk factor for progression to vaginal cancer [28].

Unlike other authors [22], we did not find any correlation between age at diagnosis and the histological grade of VAIN. However, Zhou et al. also reported a poor rank correlation [22], whereas Boonlikit et al. did not show any significant distribution of patients’ age among different VAIN grade groups [17]. The mean age of our patients was 52.4 ± 12.8 years. Therefore, we did not investigate whether the post-menopausal status correlated with an increased risk of VAIN because of a thinner vaginal epithelium that results in more susceptibility to changes in the vaginal microbiome and HPV infections [26].

Even immunosuppression was not associated with the development of VAIN in our cohort, as opposed to previous studies [29], probably due to the small proportion of immunosuppressed patients (11.5%).

Most diagnoses of VAIN were preceded by an abnormal pap smear result, thus supporting the assumption that cytology, in combination with a HR-HPV test, is an effective tool for early diagnosis of VAIN, even after hysterectomy, since its sensitivity is not inferior to that for CIN2+ detection [5]. We did not investigate whether cytology positivity was higher in patients with a previous hysterectomy, as recently shown by Zhang et al. in a large retrospective series of VAIN. However, the combined use of cytology and HPV testing could curb this issue, since no statistically significant difference in co-testing positivity was identified in women with or without a history of previous hysterectomy [30]. HR-HPV status was known only in 160 out of 255 enrolled patients. Most of the missing data were found in women with a first diagnosis of VAIN in the early 2000s, when HPV testing was neither applied for primary screening nor routinely performed as a triage test after abnormal cytology. In our cohort with an available Cobas result, 83.8% of cases were affected by HR-HPV infection, with or without HPV 16 and 18, as reported by previous literature [26]. Most of the women affected by VAIN3 were positive for HR-HPV with 16 and/or 18 (53.5%). As already explained, this association was only borderline significant, due to the large number of missing Cobas results, but is in agreement with previous data [31]. HPV 16, 52, 56 and 58 have been identified as the most prevalent genotypes in high-grade VAIN [30], while many LR and HR genotypes have been linked to the development of low-grade VAIN. HPV type distribution is even more heterogeneous in case of coexisting cervical lesions, although a recent study by Zhang et al. showed that different HPV genotypes are independent causative agents of coexisting CIN and VAIN [32]. Furthermore, specific HPV genotypes, particularly HPV 16, have been related to a greater risk of VAIN persistence, progression and recurrence [28]. Therefore, HPV genotyping could be a useful tool for risk-stratification of patients affected by VAIN.

Regarding the diagnostic accuracy of colposcopy in relation to the histological grade of VAIN, our study confirmed that colposcopic grade G2 and vascularity were significantly associated with VAIN3, including VAIN3 with stromal microinvasion. These associations have been widely demonstrated by other authors [17,19,20], that already observed specific abnormal colposcopic findings, such as grade 2 and vascular punctuation, more commonly in women diagnosed with VAIN3 rather than with VAIN2 or VAIN1. Interestingly, our study included a larger proportion of women diagnosed with VAIN3, when compared to previous studies, and also considered microinvasive VAIN3 as a separate category [17,19]. Moreover, in our cohort we found colposcopic grade 2 in 46.6% of women, that is a prevalence roughly double that previously reported by Sopracordevole et al. (22.7%) [19].

The correlation between vascularity and high-grade VAIN has been already explained by Boonlikit et al., as a consequence of the lack of vascular structure in very mature squamous vaginal epithelium. Thus, vascular patterns appear later, as distinct from the cervical dysplastic process, in which vascular punctuation appears early due to the immature squamous metaplasia of the transformation zone [17].

Conversely, our results showed a significant association between papillary lesions and VAIN3, and in particular, microinvasive VAIN3. This is totally different from the evidence of other authors who detected micropapillary patterns more frequently in women affected by low-grade VAIN [19,20,22]. The exact meaning of this colposcopic feature is still unclear and lacking. According to our experience, if the papillary pattern is caused by a persistent HPV infection, as already suggested [19,20,22], it should be considered as the expression of dysplastic progression towards high-grade VAIN. Another possible explanation of this relevant difference could derive from the absence of this specific feature in the 2011 IFCPC colposcopic terminology [21]. In fact, a recent study found poor concordance between the diagnosis based on the 2011 IFCPC colposcopic classification and vaginal histology for the high-grade VAIN category (only 35.71%), with a substantial false negative rate (42.86%), thus suggesting that the IFCPC nomenclature should be improved and better standardized for vaginal lesions [22].

Notably, we did not find any significant difference among VAIN grade groups regarding lesions number, as against that sustained by Zhou et al. [20]. Even vaginal localization was not significantly associated with the histological grade of VAIN. Nevertheless, the prevalence of VAIN in the vaginal vault (38.7%) and in the upper third of vaginal walls (45.1%) was much higher than in the lower two thirds (16.2%), in agreement with previously reported frequencies [3,22,33,34].

To the best of our knowledge, this is the first study to investigate and portray colposcopic characteristics of not only low- and high-grade VAIN, but also VAIN3 with stromal microinvasion, that should always be correctly identified before choosing a therapeutic approach.

The main strengths of the study are related to the higher proportion of high-grade VAIN in our population and the data homogeneity, because all colposcopies were performed at a referral oncologic center, only by trained colposcopists, with particular expertise in the diagnosis and treatment of vaginal lesions. On the contrary, limits of the study include selection bias related to the single center retrospective design of the study and the amount of missing data in old medical reports.

A better defined and standardized application of the 2011 ICFPC colposcopic terminology for vaginal lesions could be useful for correct diagnosis and management of VAIN. Indeed, identifying risk factors and colposcopic patterns predictive for high-grade VAIN would help the colposcopist to sample the area most likely to contain VAIN3 or stromal invasion, especially in large and multifocal lesions, which could simultaneously hold different grades of VAIN.

Appropriate diagnosis of VAIN3, with or without stromal microinvasion, is mandatory to choose the optimal management, which still remains challenging and controversial for high-grade VAIN. Several therapeutic regimens, including conservative surveillance, ablative procedures and surgical excisions, have been proposed over time, due to a high recurrence rate of VAIN2-3 despite the type of treatment [4,35,36,37,38]. Hence, proper and accurate diagnosis could allow for more personalized risk-based management, based on risk factors, colposcopic patterns and the histologic grade of VAIN.

## 5. Conclusions

The risk of high-grade VAIN should not be underestimated in women with a history of current or former smoking and previous hysterectomy for CIN2+, which also represents a risk factor for recurrence and progression to vaginal cancer. Colposcopic findings, including grade 2, papillary and vascular patterns, are predictive factors for VAIN3 with or without stromal microinvasion. Accurate colposcopic and histologic diagnosis is crucial for the optimal management of vaginal pre-cancers and cancers. In addition, HPV genotyping could be a helpful tool for risk stratification and prompt identification of women with VAIN3 at higher risk of persistence, progression and recurrence.

## Figures and Tables

**Table 1:** Patients’ characteristics summary statistics ^a^ by VAIN grade at diagnosis.

Characteristic	Category	VAIN Grade				
		All Patients *n* = 255	VAIN 1 *n* = 52	VAIN 2 *n* = 55	VAIN 3 *n* = 148	*p*-Value
Age (years) at first diagnosis		52.4 (12.8)	51.4 (12.2)	50.1 (13.4)	53.7 (12.7)	0.18
Current/former smoker		86 (34.1)	18 (34.6)	27 (50.9)	41 (27.9)	0.01
Parity		129 (55.4)	21 (42.9)	21 (46.7)	87 (62.6)	0.02
Immunosuppression		29 (11.5)	5 (9.6)	5 (9.1)	19 (13.0)	0.77
Hormonal therapy		40 (15.7)	8 (15.4)	7 (12.7)	25 (16.9)	0.82
Previous hysterectomy	CIN2+	97 (38.0)	13 (25.0)	17 (30.9)	67 (45.3)	
	Other	14 (5.5)	3 (5.8)	6 (10.9)	5 (3.4)	
	No hysterectomy	144 (56.5)	36 (69.2)	32 (58.2)	76 (51.4)	0.02
Previous cervical cancer	Yes	56 (22.1)	12 (23.5)	6 (10.9)	38 (25.9)	
	No/Other tumors ^b^	197 (77.9)	39 (76.5)	49 (89.1)	109 (74.2)	0.06
Previous CIN	No/CIN1	150 (59.3)	33 (63.5)	31 (56.4)	86 (58.9)	
	CIN2-3	103 (40.7)	19 (44.7)	24 (43.6)	60 (41.1)	0.75
Concomitant CIN	No/CIN1	224 (87.8)	48 (92.3)	47 (85.5)	129 (87.2)	
	CIN2-3	31 (12.2)	4 (7.7)	8 (14.6)	19 (12.8)	0.52
Previous VIN	No	232 (91.7)	47 (92.2)	51 (92.7)	134 (91.2)	
	VIN3	21 (8.3)	4 (7.8)	4 (7.3)	13 (8.8)	1.00
Concomitant VIN	No	233 (91.4)	48 (92.3)	51 (92.7)	134 (90.5)	
	VIN1	4 (1.6)	2 (3.9)	0	2 (1.4)	
	VIN2	1 (0.4)	0	1 (1.8)	0	
	VIN3	17 (6.7)	2 (3.9)	3 (5.5)	12 (8.1)	0.34
Previous AIN	No	247 (97.6)	49 (96.1)	53 (96.4)	145 (98.6)	
	AIN2	1 (0.4)	0	1 (1.8)	0	
	AIN3	5 (2.0)	2 (3.9)	1 (1.8)	2 (1.4)	0.28
Concomitant AIN	No	253 (99.6)	51 (98.1)	54 (100)	148 (100)	
	AIN1	1 (0.4)	1 (1.9)	0	0	0.20
HR-HPV	HR+ with 16 and/or 18	71 (44.4)	11 (30.6)	14 (36.8)	46 (53.5)	
	HR+ without 16 and 18	63 (39.4)	14 (36.8)	20 (52.6)	26 (30.2)	
	Negative	26 (16.3)	8 (22.2)	4 (10.5)	14 (16.3)	0.05
Cytology	Negative	7 (3.1)	2 (4.1)	2 (4.0)	3 (2.3)	
	ASCUS-LSIL	67 (29.3)	29 (59.2)	15 (30.0)	23 (17.7)	
	ASCH-HSIL	145 (63.3)	17 (34.7)	33 (66.0)	95 (73.1)	
	SCC	10 (4.4)	1 (2.0)	0	9 (6.9)	<0.001

^a^*n* (column %) for categorical variable, Mean (SD) for Age; SD = Standard deviation; ^b^ including 153 women with no history of any cancer and 44 women with a previous history of other non-HPV-related tumors (i.e., breast cancer, Hodgkin’s lymphoma, colorectal cancer, endometrial cancer); VAIN = Vaginal Intraepithelial Neoplasia; CIN = Cervical Intraepithelial Neoplasia; VIN = Vulvar Intraepithelial Neoplasia; AIN = Anal Intraepithelial Neoplasia; HR-HPV = High-risk Human Papillomavirus; ASCUS = Atypical Squamous Cells of Undetermined Significance; LSIL = Low Squamous Intraepithelial Lesion; ASCH = Atypical Squamous Cells cannot exclude HSIL; HSIL = High Squamous Intraepithelial Lesion; SCC = Squamous Cell Carcinoma.

**Table 2:** Patients’ colposcopic features summary statistics ^a^ by VAIN grade at diagnosis.

Characteristic	Category	VAIN Grade				
		All Patients *n* = 255	VAIN 1 *n* = 52	VAIN 2 *n* = 55	VAIN 3 ^b^ *n* = 148	*p*-Value
Grade	G1	134 (53.4)	45 (88.2)	41 (74.6)	48 (33.1)	
	G2	117 (46.6)	6 (11.8)	14 (25.4)	97 (66.9)	<0.001
Lesion type	Flat	145 (59.2)	41 (80.4)	38 (70.4)	66 (47.1)	
	Papillary	100 (40.8)	10 (19.6)	16 (29.6)	74 (52.9)	<0.001
Multifocality	Unifocal	141 (56.6)	34 (65.4)	29 (53.7)	78 (54.6)	
	Multifocal	108 (43.4)	18 (34.6)	25 (46.3)	65 (45.4)	0.36
Vascular pattern	No	196 (80.3)	51 (98.1)	51 (92.7)	94 (68.6)	
	Yes	48 (19.7)	1 (1.9)	4 (7.3)	43 (31.4)	<0.001
Vaginal localization	Vault	98 (38.7)	15 (28.9)	21 (38.2)	62 (42.5)	
	Upper third	114 (45.1)	24 (46.2)	24 (43.6)	66 (45.2)	
	Middle third	26 (10.3)	10 (19.2)	8 (14.6)	8 (5.5)	
	Lower third	15 (5.9)	3 (5.8)	2 (3.6)	10 (6.9)	0.09

^a^*n* (column %); ^b^ Including *n* = 10 VAIN3 with stromal microinvasion; VAIN = Vaginal Intraepithelial Neoplasia; G = Grade.

**Table 3:** Patients’ colposcopic features summary statistics ^a^ by VAIN grade at diagnosis, considering microinvasive VAIN3 as a separate category.

Characteristic	Category	VAIN					
		All Patients *n* = 255	VAIN 1 *n* = 52	VAIN 2 *n* = 55	VAIN 3 *n* = 138	VAIN 3 Microinvasive *n* = 10	*p*-Value
Grade	G1	134 (53.4)	45 (88.2)	41 (74.6)	48 (35.6)	0	
	G2	117 (46.6)	6 (11.8)	14 (25.4)	87 (64.4)	10 (100)	<0.001
Lesion type	Flat	145 (59.2)	41 (80.4)	38 (70.4)	66 (50.4)	0	
	Papillary	100 (40.8)	10 (19.6)	16 (29.6)	65 (49.6)	9 (90.0)	<0.001
Multifocality	Unifocal	141 (56.6)	34 (65.4)	29 (53.7)	76 (56.7)	2 (22.2)	
	Multifocal	108 (43.4)	18 (34.6)	25 (46.3)	58 (43.3)	7 (77.8)	0.11
Vascularity	No	196 (80.3)	51 (98.1)	51 (92.7)	89 (69.5)	5 (55.6)	
	Yes	48 (19.7)	1 (1.9)	4 (7.3)	39 (30.5)	4 (44.4)	<0.001
Vaginal localization	Vault	98 (38.7)	15 (28.9)	21 (38.2)	58 (42.7)	4 (40.0)	
	Upper third	114 (45.1)	24 (46.2)	24 (43.6)	61 (44.9)	5 (50.0)	
	Middle third	26 (10.3)	10 (19.2)	8 (14.6)	8 (5.9)	0	
	Lower third	15 (5.9)	3 (5.8)	2 (3.6)	9 (6.6)	1 (10.0)	0.23

^a^*n* (column %); VAIN = Vaginal Intraepithelial Neoplasia; G = Grade.

**Table 4:** Multivariate analysis of variance of risk factors for VAIN excluding lesion type and vascularity.

VAIN Grade	Factor	Level	OR (95% CI)	*p*-Value
VAIN2 vs. VAIN1	Smoking	No	Ref	
		Yes	1.53 (0.62,3.78)	0.36
	Previous hysterectomy	No hysterectomy	Ref	
		CIN2+	1.20 (0.43,3.38)	0.73
		Other	1.11 (0.19,6.50)	0.91
	Colposcopic Grade	G1	Ref	
		G2	4.77 (1.40,16.2)	0.01
VAIN3 ^a^ vs. VAIN1	Smoking	No	Ref	
		Yes	0.61 (0.25,1.47)	0.27
	Previous hysterectomy	No hysterectomy	Ref	
		CIN2+	2.15 (0.89,5.24)	0.09
		Other	1.46 (0.28,7.64)	0.66
	Colposcopic Grade	G1	Ref	
		G2	20.4 (6.67,61.4)	<0.001

^a^ Including *n* = 10 VAIN3 with stromal microinvasion; VAIN = Vaginal Intraepithelial Neoplasia; OR = Odds Ratio; CI = Confidence Interval; CIN = Cervical Intraepithelial Neoplasia; G = Grade.

**Table 5:** Multivariate analysis of variance of risk factors for VAIN excluding colposcopic grade.

VAIN Grade	Factor	Level	OR (95% CI)	*p*-Value
VAIN2 vs. VAIN1	Smoking	No	Ref	
		Yes	1.49 (0.60,3.67)	0.40
	Previous hysterectomy	No hysterectomy	Ref	
		CIN2+	1.27 (0.45,3.56)	0.65
		Other	0.91 (0.15,5.69)	0.92
	Lesion type	Flat	Ref	
		Papillary	2.90 (1.07,7.89)	0.03
	Vascularity	No	Ref	
		Yes	2.81 (0.25,31.5)	0.40
VAIN3 ^a^ vs. VAIN1	Smoking	No	Ref	
		Yes	0.79 (0.35,1.78)	0.57
	Previous hysterectomy	No hysterectomy	Ref	
		CIN2+	2.37 (1.02,5.36)	0.04
		Other	1.04 (0.20,5.36)	0.96
	Lesion type	Flat	Ref	
		Papillary	4.33 (1.79,10.5)	0.001
	Vascularity	No	Ref	
		Yes	14.4 (1.86,112)	0.01

^a^ Including *n* = 10 VAIN3 with stromal microinvasion; VAIN = Vaginal Intraepithelial Neoplasia; OR = Odds Ratio; CI = Confidence Interval; CIN = Cervical Intraepithelial Neoplasia.

## Data Availability

The data presented in this study are available on request from the corresponding author. The data are not publicly available due to patients’ privacy restrictions. The data are safely stored in a private database of the European Institute of Oncology, Milan, Italy.

## Supplementary Materials

The following supporting information can be downloaded at: https://www.mdpi.com/article/10.3390/diagnostics13020176/s1, Table S1: Patients’ colposcopic features summary statisticsa by VAIN grade at diagnosis; Table S2: Missing data distribution by VAIN grade at diagnosis; Table S3: Colposcopic Grade association with Lesion type and Vascularity.
