# Supplementary material for: Which Risk Factors and Colposcopic Patterns Are Predictive for High-Grade VAIN? A Retrospective Analysis
Source: Diagnostics (Basel). 2023 Jan 4;13(2):176. doi: 10.3390/diagnostics13020176 (PMC9858341; doi:10.3390/diagnostics13020176)
Supplement: Supplementary file 1 [file diagnostics-13-00176-s001.zip › diagnostics-2035410-Supplementary Materials.pdf]

diagnostics

## Supplementary Tables

**Table S1.** Patients' colposcopic features summary statistics[a] by VAIN grade at diagnosis.

| Characteristic | | All patients N = 245 | VAIN Grade VAIN 1 N = 52 | VAIN 2 N = 55 | VAIN 3 [b] N = 138 | p-value |
|---|---|---|---|---|---|---|
| Grade | G1 | 134 (55.6) | 45 (88.2) | 41 (74.6) | 48 (35.6) | |
| | G2 | 107 (44.4) | 6 (11.8) | 14 (25.4) | 87 (64.4) | **< 0.001** |
| Lesion type | Flat | 145 (61.4) | 41 (80.4) | 38 (70.4) | 66 (50.4) | |
| | Papillary | 91 (38.6) | 10 (19.6) | 16 (29.6) | 65 (49.6) | **< 0.001** |
| Multifocality | Unifocal | 139 (57.9) | 34 (65.4) | 29 (53.7) | 76 (56.7) | |
| | Multifocal | 101 (42.1) | 18 (34.6) | 25 (46.3) | 58 (43.3) | 0.44 |
| Vascularity | No | 191 (81.3) | 51 (98.1) | 51 (92.7) | 89 (69.5) | |
| | Yes | 44 (18.7) | 1 (1.9) | 4 (7.3) | 39 (30.5) | **< 0.001** |
| Vaginal localization | Vault | 94 (38.7) | 15 (28.9) | 21 (38.2) | 58 (42.7) | |
| | Upper third | 109 (44.9) | 24 (46.2) | 24 (43.6) | 61 (44.9) | |
| | Middle third | 26 (10.7) | 10 (19.2) | 8 (14.6) | 8 (5.9) | |
| | Lower third | 14 (5.8) | 3 (5.8) | 2 (3.6) | 9 (6.6) | 0.13 |

[a] N (column %); [b] Excluding N = 10 VAIN3 with stromal microinvasion; VAIN = Vaginal Intraepithelial Neoplasia.

**Table S2.** Missing data distribution[a] by VAIN grade at diagnosis.

| Characteristic | All patients N = 255 | VAIN VAIN 1 N = 52 | VAIN 2 N = 55 | VAIN 3 [b] N = 148 | p-value |
|---|---|---|---|---|---|
| Current/former smoker | 3 (1.2) | 0 | 2 (3.6) | 1 (0.7) | 0.23 |
| Parity | 22 (8.6) | 3 (5.8) | 10 (18.2) | 9 (6.1) | **0.03** |
| Immunosuppression | 2 (0.8) | 0 | 0 | 2 (1.4) | 1.00 |
| Previous cancer | 2 (0.8) | 1 (1.9) | 0 | 1 (0.7) | 0.41 |
| Previous CIN | 2 (0.8) | 0 | 0 | 2 (1.4) | 1.00 |
| Previous VIN | 2 (0.8) | 1 (1.9) | 0 | 1 (0.7) | 0.41 |
| Previous AIN | 2 (0.8) | 1 (1.9) | 0 | 1 (0.7) | 0.41 |
| Concomitant AIN | 1 (0.4) | 0 | 1 (1.8) | 0 | 0.42 |
| HR-HPV | 95 (37.3) | 16 (30.8) | 17 (30.9) | 62 (41.9) | 0.21 |
| Cytology | 26 (10.2) | 3 (5.8) | 5 (9.1) | 18 (12.2) | 0.45 |
| Vaginal localization | 2 (0.8) | 0 | 0 | 2 (1.4) | 1.00 |
| Grade | 4 (1.6) | 1 (1.9) | 0 | 3 (2.0) | 0.65 |
| Lesion type | 10 (3.9) | 1 (1.9) | 1 (1.8) | 8 (5.4) | 0.49 |
| Multifocality | 6 (2.4) | 0 | 1 (1.8) | 5 (3.4) | 0.63 |
| Vascularity | 11 (4.3) | 0 | 0 | 11 (7.4) | **0.02** |

[a] N (column %); [b] Including N = 10 VAIN3 with stromal microinvasion; VAIN = Vaginal Intraepithelial Neoplasia; CIN = Cervical Intraepithelial Neoplasia; VIN = Vulvar Intraepithelial Neoplasia; AIN = Anal Intraepithelial Neoplasia; HR-HPV = High-risk Human Papillomavirus.

**Table S3.** Colposcopic Grade association with Lesion type and Vascularity.

| | Colposcopic Grade, N (column %) G1 | G2 | p-value |
|---|---|---|---|
| Lesion type | | | |
| Flat | 118 (89.4) | 27 (24.6) | |
| Papillary | 14 (10.6) | 83 (75.5) | **< 0.001** |
| Vascularity | | | |
| No | 129 (97.0) | 65 (60.2) | |
| Yes | 4 (3.0) | 43 (39.8) | **< 0.001** |
